# AI-Assisted Identification of Primary and Secondary Metabolomic Markers for Postoperative Delirium

**DOI:** 10.3390/ijms252111847

**Published:** 2024-11-04

**Authors:** Vladimir A. Ivanisenko, Artem D. Rogachev, Aelita-Luiza A. Makarova, Nikita V. Basov, Evgeniy V. Gaisler, Irina N. Kuzmicheva, Pavel S. Demenkov, Artur S. Venzel, Timofey V. Ivanisenko, Evgenia A. Antropova, Nikolay A. Kolchanov, Victoria V. Plesko, Gleb B. Moroz, Vladimir V. Lomivorotov, Andrey G. Pokrovsky

**Affiliations:** 1 Institute of Cytology and Genetics, Siberian Branch of Russian Academy of Sciences (SB RAS), Novosibirsk 630090, Russia; makarovaaa@bionet.nsc.ru (A.-L.A.M.); demps@bionet.nsc.ru (P.S.D.); venzel@bionet.nsc.ru (A.S.V.); itv@bionet.nsc.ru (T.V.I.); nzhenia@bionet.nsc.ru (E.A.A.); kol@bionet.nsc.ru (N.A.K.); 2 The Artificial Intelligence Research Center of Novosibirsk State University, Novosibirsk 630090, Russia; artrogachev@yandex.ru (A.D.R.); basov@nioch.nsc.ru (N.V.B.); evgeniy.gaysler@mail.ru (E.V.G.); i.kuzmicheva1@g.nsu.ru (I.N.K.); 3 Kurchatov Genomic Center of Institute of Cytology and Genetics, SB RAS, Novosibirsk 630090, Russia; 4 Department of Information Biology, Novosibirsk State University, Novosibirsk 630090, Russia; 5 N. N. Vorozhtsov Novosibirsk Institute of Organic Chemistry, Siberian Branch, Russian Academy of Sciences, Novosibirsk 630090, Russia; 6 V. Zelman Institute for the Medicine and Psychology, Novosibirsk State University, Novosibirsk 630090, Russia; agpok@inbox.ru; 7 E. Meshalkin National Medical Research Center, Novosibirsk 630055, Russia; vika-artemeva@mail.ru (V.V.P.); glebmorozz@gmail.com (G.B.M.); v.lomivorotov@gmail.com (V.V.L.); 8 Penn State Milton S. Hershey Medical Center, Hershey, PA 17033, USA

**Keywords:** postoperative delirium, metabolomics, neural networks, gene networks, biomarkers

## Abstract

Despite considerable investigative efforts, the molecular mechanisms of postoperative delirium (POD) remain unresolved. The present investigation employs innovative methodologies for identifying potential primary and secondary metabolic markers of POD by analyzing serum metabolomic profiles utilizing the genetic algorithm and artificial neural networks. The primary metabolomic markers constitute a combination of metabolites that optimally distinguish between POD and non-POD groups of patients. Our analysis revealed L-lactic acid, inositol, and methylcysteine as the most salient primary markers upon which the prediction accuracy of POD manifestation achieved AUC = 99%. The secondary metabolomic markers represent metabolites that exhibit perturbed correlational patterns within the POD group. We identified 54 metabolites as the secondary markers of POD, incorporating neurotransmitters such as gamma-aminobutyric acid (GABA) and serotonin. These findings imply a systemic disruption in metabolic processes in patients with POD. The deployment of gene network reconstruction techniques facilitated the postulation of hypotheses describing the role of established genomic POD markers in the molecular-genetic mechanisms of metabolic pathways dysregulation, and involving the identified primary and secondary metabolomic markers. This study not only expands the understanding of POD pathogenesis but also introduces a novel technology for the bioinformatic analysis of metabolomic data that could aid in uncovering potential primary and secondary markers in diverse research domains.

## 1. Introduction

Postoperative delirium (POD) is an acute state typified by cognition and attention deficits, disorganized thoughts, and disorientation, and is a frequent implication of assorted surgical procedures. Accumulating evidence associates POD with detrimental outcomes, such as prolonged hospital stays, augmented risk of complications, and escalated mortality rates [1]. Recent studies have demonstrated that the occurrence of POD is not only a marker of immediate postoperative complications but also has long-term implications for patient outcomes [2]. For instance, delirium following coronary artery bypass graft surgery is associated with increased late mortality, emphasizing POD’s significant impact on patient survival even years after the initial episode [3]. POD substantially affects the quality of life of patients. Persistent cognitive impairment and functional decline are commonly observed in patients who have experienced delirium, leading to reduced independence and an increased need for long-term care [4].

Economically, POD imposes substantial costs on healthcare systems due to extended hospital stays, increased need for postoperative care, and higher rates of rehospitalization. Leslie and Inouye [5] estimated that delirium is associated with over $143 billion in annual healthcare expenditures in the United States, reflecting both direct medical costs and indirect costs such as lost productivity and caregiver burden. The economic implications underscore the importance of early detection, prevention, and management strategies to mitigate the impact of POD on patients and health systems.

The propensity to POD manifestation escalates concurrently with age, as exemplified by a study reporting a 52% incidence of POD in cardiac surgery patients aged 60 years or older [6]. Risk factors for POD also include duration of general anesthesia during surgery, neurotransmitter imbalance, neuroinflammation, metabolic syndrome, and others [7,8]. Addressing these risk factors through tailored perioperative care can potentially reduce the incidence and severity of POD, improving patient outcomes and reducing healthcare costs.

Metabolomics, by providing a snapshot of current biochemical activity, has been instrumental in diverse areas, such as profiling disease biomarkers [9,10], monitoring disease progression [11], elucidating xenobiotic metabolism [12], and assessing drug toxicity [13]. This sets metabolomics apart from genomics and proteomics, which only offer potential scenarios for the condition’s development [14].

The levels of individual metabolites and the interplay between various metabolites can serve as indicators of physiological and pathological transformations within an organism [15]. The stratification of metabolic profiles into predefined classes based on individual metabolites or the sets of metabolites forms the crux of extant computational biomarker discovery methods [16]. Such indicators, referred to as primary biomarkers, could significantly contribute to diagnostic and therapeutic strategies.

Perturbations in the interplay among metabolites can offer insightful explanations of metabolic pathway dysfunction across an array of pathologies at a systemic level [17]. Metabolites that exhibit disrupted coordinated variability with their counterparts may be designated as secondary biomarkers [18,19]. These secondary biomarkers cannot only expand our knowledge of the molecular mechanisms underlying physiological or pathophysiological processes, but can also improve the predictive capacity of primary biomarkers [20].

Evaluation of metabolite levels and analysis of the correlations between metabolite concentrations is an applicable strategy to encapsulate the intricate relationships between metabolites [21]. Xiao et al. put forward a method designed to analyze a network of pairwise correlations among metabolites, premised on the Generalized Singular Value Decomposition (GSVD) algorithm, computed separately for POD and non-POD groups [22]. This approach enables the discovery of clusters of metabolites predicated on mutual correlations that differ between patient and control groups. Correlation analysis has also been exploited to establish the linkage between metabolites and phospholipid species, and is grounded in metabolomic and lipidomic data derived from prostate cancer tissue [23].

In previous investigations, our team executed liquid chromatography with tandem mass spectrometry (LC–MS/MS) analysis of cerebrospinal fluid (CSF) and blood plasma samples collected from patients afflicted with high-grade glioma [24]. Consequently, we uncovered correlations between the metabolic profiles of blood plasma and CSF.

The identification of interrelated metabolites from the analysis of metabolomic data is challenging due to the systemic control of metabolic processes. Generally, the pairwise correlations observed between metabolites are insignificant, even when the pairs are part of the same metabolic pathway [25,26,27]. This phenomenon could be attributed to the inherent stochasticity of metabolic processes [28,29,30,31]. Thus, explicit relationships between metabolites may be hidden, adding another layer of complexity to the analysis.

Machine learning techniques, notably those encompassing artificial intelligence, are increasingly being harnessed for the analysis of high-dimensional omics data [32]. Autoencoders (AEs) are a class of unsupervised neural network architectures devised for dimensionality reduction that can capture nonlinear relationships [33]. Autoencoder structure typically contains an input layer of neurons, one or more hidden layers, and an output layer, replicating the input data. The goal of the autoencoder is to minimize the reconstruction error between input and output data.

Autoencoders have been demonstrated to adeptly manage the dimensionality reduction of original data by establishing nonlinear relationships between features in the input data. Several variants of autoencoder architecture have been devised, such as convolutional, regularized, variational, sparse, stacked, deep, and generative [34]. In particular, variational autoencoder-derived latent representations of metabolomic data have been employed to analyze the groups of patients with conditions such as type 2 diabetes, acute myeloid leukemia, and schizophrenia [35]. Likewise, a supervised autoencoder (SAE) approach has been utilized for the classification of clinical metabolomic data [36].

Denoising autoencoders are a subclass of neural network models that are a viable method for generating lower-dimensionality latent representations of salient information [37]. For instance, a deep learning-empowered denoising autoencoder has been deployed to gauge peak quality in liquid chromatography–high resolution mass spectrometry (LC−MS) data, as well as to forecast peaks by eliminating noise from the original peak profiles [38]. Similarly, a denoising autoencoder (SERDA) has been used to rectify errors in large-scale metabolomic data generated via gas chromatography–mass spectrometry [39]. Furthermore, a normalization autoencoder (NormAE), a brainchild of nonlinear AEs and adversarial learning, has been utilized to expunge batch effects in LC–MS-based metabolomics data [40]. Additionally, a number of methodologies for analyzing metabolic pathways and gene networks have been proposed to decode the molecular genetic mechanisms underlying observed metabolomic data. The MetaboAnalyst 5.0 web tool, for instance, provides a comprehensive toolkit for the analysis of metabolomic data in the context of metabolic pathways [41].

Gene networks can confer valuable insights regarding genetic regulation of identified metabolic pathways, thereby forming the basis for the integration of metabolomic and genomic data. Previously, we designed the ANDSystem, a computer tool for the reconstruction of gene networks, based on the automated extraction of knowledge from scientific publications and factual databases [42]. The ANDSystem has been applied to diverse problems, ranging from the reconstruction of host–virus interaction gene networks [43,44], gene prioritization [45,46], and to identify new potential pharmacological targets [47]. Notably, utilizing the ANDSystem to evaluate serum metabolomic profiles derived from COVID-19 patients facilitated the identification of potential regulatory pathways and the key SARS-CoV-2 proteins implicated in the disruption of metabolic processes during the course of infection [48].

In this study, we performed a metabolomic analysis of blood plasma acquired from two distinct patient groups who underwent cardiac surgery. One group developed postoperative delirium, whilst the other group did not exhibit this complication. Blood samples were procured prior to surgery as we examined the data regarding preoperative metabolomic profiles. The metabolomic profiles derived from these samples revealed potential primary and secondary biomarkers.

The primary biomarkers were identified as composite metabolites that markedly differentiated patients with postoperative delirium from those without this condition. The genetic algorithm was employed to discern these combinations and several groups consisting of three to four metabolites demonstrated a comparable ability to distinguish patients exhibiting delirium from those who did not. Remarkably, two metabolites, L-lactic acid and inositol, were present in nearly all combinations, with inclusion rates of 100% and 95%, respectively. Other metabolites, methylcysteine and Adenine, were included in 40% and 12% of combinations, respectively. In total, 37 distinct metabolites were involved in the combinations.

For identification of the secondary markers, we suggest a method predicated on the detection of anomalies within data, utilizing a denoising autoencoder [49]. In general, utilizing autoencoders for anomaly detection has been substantiated by multiple studies [49,50,51,52,53]. The secondary markers represent metabolites, for which the denoising autoencoder trained on metabolomic profiles from non-POD patients failed to replicate metabolite concentrations for patients in the POD group. Such aberrations in the autoencoder’s outputs can be attributed to the disruption of encoded intricate nonlinear relationships between metabolite concentrations. These relationships allow us to reduce the dimensionality of metabolomic profile input within the neural network’s hidden layer without substantial loss of prediction accuracy in the output.

We herein suggest a novel approach to identifying the secondary metabolomic biomarkers, deploying a digital patient model predicated on the use of a denoising autoencoder, applied to the metabolomic data. The autoencoder detects metabolites whose interconnections with other metabolites, specific to the non-POD group, were disrupted in patients manifesting delirium. We identified 54 metabolites, underscoring the systemic nature of metabolic aberrations in delirium patients. Interestingly, there were overlaps between the primary and secondary biomarkers, as evidenced by eight shared metabolites, including inositol, Gamma-Aminobutyric Acid (GABA), biotin, and others. Of particular interest, the primary metabolomic markers of POD included neurotransmitters such as GABA, serotonin, and its precursors L-Tryptophan and 5-Hydroxy-L-tryptophan. Given that some metabolites do not cross the blood–brain barrier (BBB), we postulate that the BBB’s permeability may be compromised due to neuroinflammatory processes in patients with POD. Notably, the outflow of CSF serotonin into blood plasma was insufficient for its classification as a primary marker, yet it was significant enough for inclusion among the secondary markers.

Upon performing an overrepresentation analysis of the Kyoto Encyclopedia of Genes and Genomes (KEGG) metabolic pathways, based on the entire list of primary biomarker metabolites, no statistically significant results were obtained. Nevertheless, when the primary and secondary biomarkers were combined, metabolic pathways registering the lowest *p*-values for primary biomarkers, such as “Tryptophan metabolism” and “Pyrimidine metabolism”, emerged as statistically significant. Consequently, the secondary biomarkers amplified the statistical significance of enriched metabolic pathways, as determined by analysis of the primary markers.

Utilizing the ANDSystem for gene network reconstruction, we inferred what the potential roles of established POD genetic markers in the regulation of the identified metabolic pathways were. Several genetic markers of POD, including IFNG, TNFA, LEP, and IL6, had a substantial regulatory impact on metabolic pathways.

## 2. Results

### 2.1. Preliminary Statistical Analysis

Following the preprocessing of the LC–MS/MS metabolomic data from the preoperative blood plasma samples, 210 metabolites were selected for evaluation (Supplementary Table S1). Based on post-surgical observations, all patients were classified into POD and non-POD groups. An initial comparative statistical analysis was carried out on the obtained metabolomic data of both patient groups for each of the 210 metabolites using the Mann–Whitney test (Supplementary Table S2). This analysis failed to reveal statistically significant differences between the two patient groups. The lack of detection of individual metabolomic markers could potentially be attributed to the complexity of the molecular genetic mechanisms underlying a predisposition to POD, which likely involve the interplay of multiple metabolites.

### 2.2. Identification of the Primary Metabolomic Markers

Due to the lack of capacity of the basic statistical analysis to identify individual metabolomic markers, we next employed methods that could facilitate the identification of not just distinct metabolites, but their combinations. For this purpose, we utilized a genetic algorithm method, as described by Lu et al. [54]. The fitness of the individual in this method was assessed using an XGBoost model, which was provided with different combinations of metabolite concentrations generated by the “mutations” and “recombinations” facilitated by the genetic algorithm. This strategy yielded combinations of metabolites that optimally differentiated between patients with and without POD based on the concentration values of these metabolites. Each of these combinations comprised three to four metabolites. The accuracy of all combinations, as estimated via cross-validation, was found to be 83.3%.

**Figure 1:**
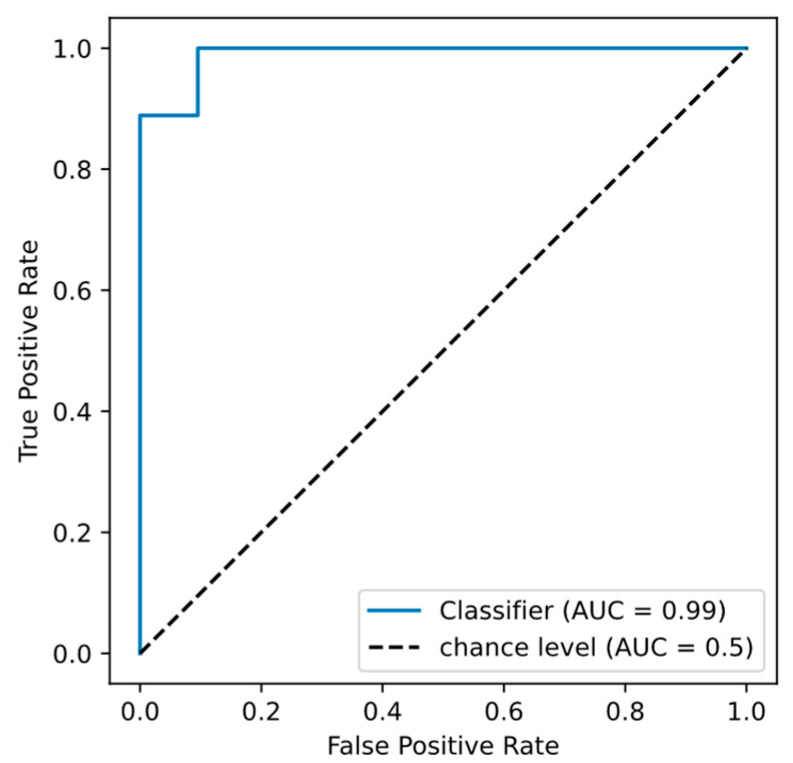
Classification accuracy for distinguishing between postoperative delirium (POD) and non-POD patient groups.

The aggregated list of metabolites encompassed within these combinations amounted to 37 unique entities (Supplementary Table S3). It is noteworthy that L-lactic acid was a constituent in all combinations, while inositol featured in 95%, methylcysteine in 40%, and Adenine in a mere 12%. The area under the ROC curve (AUC) for the metabolite triad of L-lactic acid, inositol, and methylcysteine was 0.989 (Figure 1). Consequently, these metabolites may be considered as pivotal biomarkers for the onset of POD.

We examined overrepresented KEGG metabolic pathways associated with the consolidated list of primary biomarkers using MetaboAnalyst 5.0. The KEGG pathways “Pyrimidine metabolism”, “Tryptophan metabolism”, and “Purine metabolism” were enriched with primary markers, however, the FDR values were insignificant (Table 1).

The enrichment analysis aims to identify metabolic pathways that exhibit systemic disturbances. However, indicators of the metabolic pathway disruptions can comprise not only increased or decreased concentrations of the individual metabolites, but also the disturbances of coordination or correlation between the concentrations of metabolites implicated in the metabolic pathway [14]. Metabolites exhibiting disrupted correlations with other metabolites in the context of a pathological process may be termed as the secondary metabolomic markers [16].

### 2.3. Identification of the Secondary Metabolomic Markers

To disclose the secondary metabolomic markers, we utilized an anomaly detection methodology via denoising autoencoder models, as delineated by Sakurada et al. [49]. This application of an autoencoder, inherently proficient at deciphering multiple nonlinear correlations amongst features, holds a distinct advantage over the linear pairwise correlation methods such as principal component analysis (PCA). The topology of an autoencoder includes the input layer, hidden layers, and the output layer, in which neuron values reconstruct the input neuron values. We herein employed a three-layer denoising autoencoder. The metabolite concentrations in the non-POD patient group served as the input data for autoencoder training.

We further adopted the strategy suggested by Sakurada et al. [49] to identify the secondary markers. This involved training the autoencoder model on the established dataset, followed by the model’s application to a new dataset harboring anomalies that were absent in the training set. Features were considered as anomalous if their reconstruction employing a denoising autoencoder yielded significant discrepancies in the output layer values compared to the input layer values. We examined various values for the number of neurons in the hidden layer (Supplementary Table S4). A total of 100 autoencoder models were trained using different parameter values. The trained models were subsequently applied to the metabolomic profile data of POD patients. Metabolites whose concentrations were predicted by autoencoder with statistically significant deviations from the input data were classified as the secondary markers.

Despite achieving nearly identical training accuracy across the different parameters, application of these models yielded varying sets of secondary markers. However, certain metabolites recurred frequently. We deemed metabolites resilient to the model parameter changes as the most crucial markers. The list of metabolites, ranked by the number of autoencoder models that identified them as the secondary markers, is presented in Supplementary Table S5. We conducted an overrepresentation analysis of KEGG metabolic pathways for 26 metabolites uncovered by 100 autoencoder models, however, the FDR values were insignificant (Table 2).

Of great interest is a set of metabolites that were identified in fewer models. A list of metabolites identified by at least 97 models included serotonin and was selected as the secondary markers’ set. The enrichment analysis of KEGG metabolic pathways for the resulting list of 54 secondary markers revealed the same metabolic pathways with statistical significance (Table 3).

The overlap between the primary and secondary metabolomic markers is noteworthy, with eight metabolites identified as both primary and secondary markers (Table 4). This intriguing intersection suggests that these dual-status entities may hold a privileged position in the metabolic dysregulation associated with postoperative delirium. For instance, inositol was identified as both a primary and secondary marker and was previously proposed as a prognostic marker of POD.

According to our definition, the secondary markers are characterized by disrupted inter-metabolite correlation relationships. We hypothesized that these relationships could primarily be disrupted in the metabolic processes involving the primary metabolomic markers. Consequently, the conducted overrepresentation analysis for the combined list of primary and secondary metabolomic markers would yield more accurate identification of the metabolic pathways disrupted in patients with POD.

To validate this hypothesis, we merged the lists of primary and secondary biomarkers to further analyze overrepresented KEGG metabolic pathways. Consistent with our expectations, the overrepresentation analysis of metabolic pathways for the combined list of primary and secondary metabolomic markers revealed statistically significant results. After multiple comparisons adjustment, “Glycine, serine, and threonine metabolism”, “Aminoacyl-tRNA biosynthesis”, and others were present among the significant metabolic pathways (Table 5). It is worth noting that the “Glycine, serine, and threonine metabolism” pathway previously exhibited statistical significance in the overrepresentation analysis for the set of secondary metabolomic markers (Table 3).

In the assessed KEGG metabolic pathways, the “Glycine, serine, and threonine metabolism” pathway emerged as the most statistically significant. This pathway incorporated two primary and seven secondary markers. A comparable proportion of primary to secondary markers was discernible among the metabolites of the “Aminoacyl-tRNA biosynthesis” pathway.

### 2.4. Reconstruction of the Gene Networks of Overrepresented KEGG Metabolic Pathways Regulation by Genetic Markers of POD

At the next stage, we extracted the genetic markers of POD from existing scientific reports (Supplementary Table S6). A compilation of 45 genes that were annotated as POD markers were selected for analysis. Many of these genetic markers of POD are inflammatory factors, and their escalated levels have been documented in the plasma of POD patients in the preoperative and/or early postoperative period [55,56]. For instance, elevated plasma concentrations of interleukin 6 (IL6), tumor necrosis factor alpha (TNFA), and vascular endothelial growth factor (VEGF) have been detected in the early postoperative period in elderly patients who exhibited POD [57].

**Figure 2:**
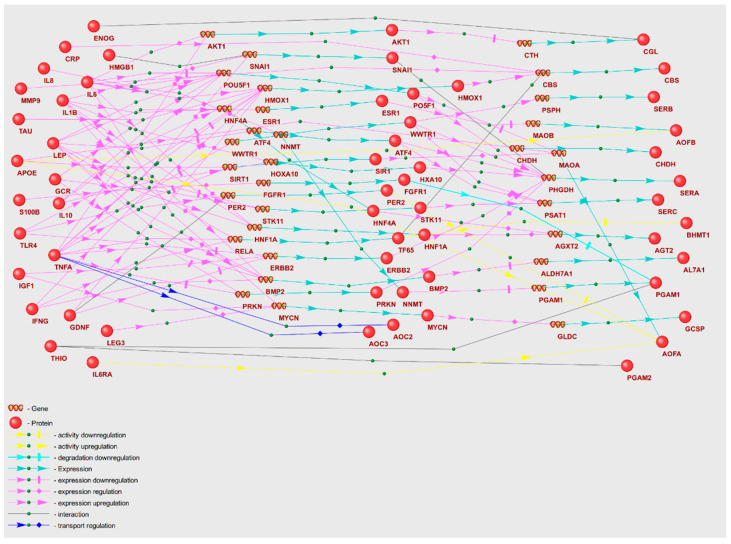
Gene network of regulation of “Glycine, serine, and threonine metabolism” enzymes by POD genetic markers.

**Figure 3:**
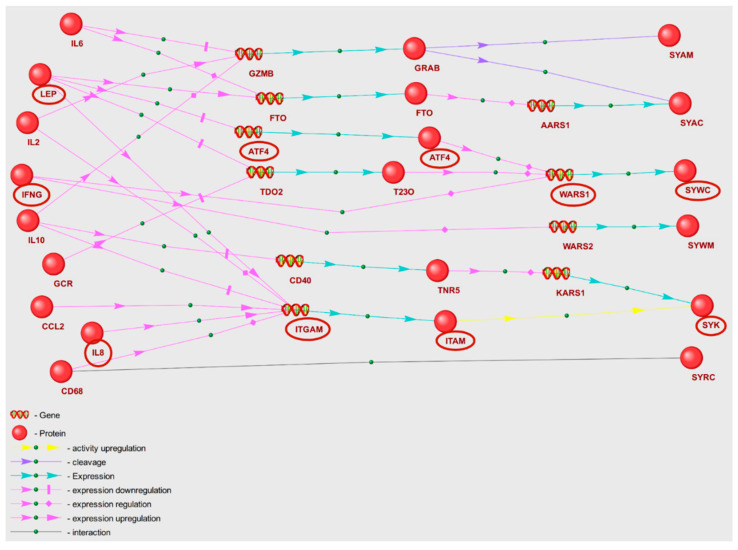
Gene network of regulation of “Aminoacyl-tRNA biosynthesis” metabolic pathway enzymes by POD genetic markers.

We proposed that POD genetic markers could play a substantial role in the mechanisms of POD pathogenesis, potentially through their involvement in metabolic pathway disruption. To validate this hypothesis, we reconstructed molecular genetic regulatory pathways using the ANDSystem. Each of the reconstructed regulatory pathways starts with proteins encoded by POD genetic markers, involves intermediate components—human proteins and genes—and ends with metabolic pathway enzymes. We considered a set of interactions between regulatory pathway participants, including regulation of gene expression, regulation of protein activity/degradation/transport, protein–protein interactions, and others. Consequently, these pathways can elucidate numerous regulatory events through which POD genetic markers could modulate the activity of metabolic pathway enzymes. We aimed to discover potential regulatory pathways for “Glycine, serine, and threonine metabolism” and “Aminoacyl-tRNA biosynthesis” by noting genetic markers of POD, as these metabolic pathways were identified as overrepresented with FDR < 0.01. The gene networks representing regulation of the enzymes of “Glycine, serine, and threonine metabolism” and “Aminoacyl-tRNA biosynthesis” metabolic pathways are illustrated in Figure 2 and Figure 3.

A graph of a gene network includes linear chains and numerous branches, as well as chain convergences that denote interactions between entities. The objects outlined in red ovals in Figure 3 illustrate the participants of the example regulatory pathways within the gene network. IL8, the genetic marker of POD, modulates the activity of tyrosine protein kinase SYK along a linear chain through upregulation of the expression of the intermediate gene ITGAM. Through a branched segment of gene network, tryptophan t-RNA ligase (WARS1) expression is directly upregulated by interferon gamma (IFNG), while leptin (LEP) modulates WARS1 expression through downregulation of intermediate gene ATF4. As a result, the gene network graphs facilitate the elucidation of the extent to which POD genetic markers exert regulatory influences on the enzymes of each of the overrepresented KEGG pathways.

Analysis of the gene networks (Figure 2 and Figure 3) reveals that 21 genetic markers regulate enzymes of the “Glycine, serine, and threonine metabolism” pathway and 9 genetic markers regulate enzymes of “Aminoacyl-tRNA biosynthesis”. Table 6 contains characteristics of the regulatory pathways, including the number of POD genetic markers that modulate enzymes involved in KEGG metabolic pathways (N1), along with the number of enzymes under regulation (N2). In Table 6, certain genetic markers are represented in more than one template for the gene network of each metabolic pathway (a single marker may be included in several templates). Among the metabolic pathways whose enzymes are most regulated, the “Glycine, serine, and threonine metabolism” pathway emerged as the most regulated. Comparable statistics can be calculated for the regulatory pathway templates. The regulation of enzymes by genetic markers is executed to the greatest extent through pathways built according to the double regulation of expression template (P4).

**Figure 4:**
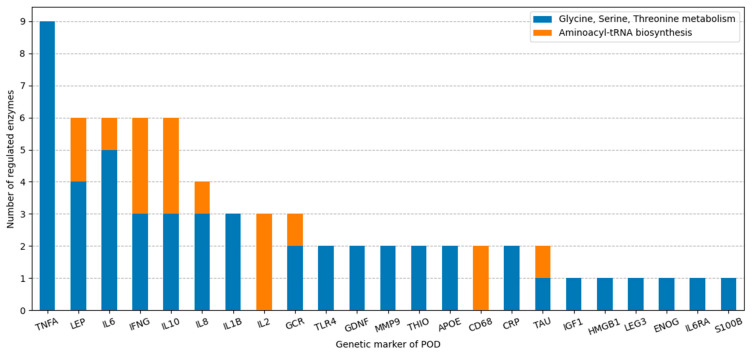
The number of regulated enzymes in KEGG metabolic pathways for each of the POD genetic markers.

In delineating the influence of distinct genetic markers on the regulation of metabolic pathways, we quantified the number of enzymes regulated by each of the genetic markers (Figure 4). It is noteworthy that 16 out of 23 markers exhibited a preferential affinity for specific metabolic pathways. For instance, TNFA specifically regulated the enzymes of the “Glycine, serine, and threonine metabolism” pathway. Alternatively, genetic markers LEP, IL6, IL10, IFNG, IL8, GCR, and TAU exerted regulation over both overrepresented metabolic pathways.

It is critical to underscore that gene regulatory networks were reconstructed using the ANDSystem, which is based on text-mining approaches, and the statistics derived may be incomplete as the reconstruction leveraged automated tools within the ANDSystem knowledge base. A characteristic of text-mining applications is their ability mostly to discern regulatory interactions that are well-studied and comprehensively documented in the literature.

### 2.5. Reconstruction of the Gene Regulatory Networks for Metabolomic Markers Not Present in Overrepresented Metabolic Pathways

It is worth noting that not all of the POD metabolomic markers identified by our study were found to participate in the overrepresented metabolic pathways. Among these are L-lactic acid and inositol, which assume significant roles as the primary markers. To detail the regulation of these metabolites by POD genetic markers, we reconstructed gene networks for regulation of the enzymes involved in lactate and myo-inositol metabolism. We retrieved enzymes implicated in lactate and myo-inositol metabolism from the KEGG database. To locate these enzymes, we queried the KEGG Pathway database (https://www.genome.jp/kegg/pathway.html, accessed on 10 November 2022) using the identifiers C00186 (lactate) and C00137 (myo-inositol) and obtained 12 and 7 metabolic pathways for these metabolites, respectively. Only human metabolic pathways with the ”hsa” prefix were considered. The search list for subsequent analysis comprised enzymes that directly catalyze reactions involving the target metabolite (myo-inositol or lactate), as well as enzymes catalyzing upstream and downstream reactions. We accounted for one to two intermediate upstream reactions and a single downstream reaction link and identified nine enzymes associated with lactate metabolism, as well as 27 enzymes associated with myo-inositol metabolism (Table 7 and Table 8). To reconstruct the regulatory pathways that elucidate the mechanisms of POD genetic marker influence on the identified enzymes, we employed the same templates that were used for analysis of the overrepresented metabolic pathways. Notably, the final links of the templates were enzymes involved in lactate or myo-inositol metabolism pathways (Table 7 and Table 8).

**Figure 5:**
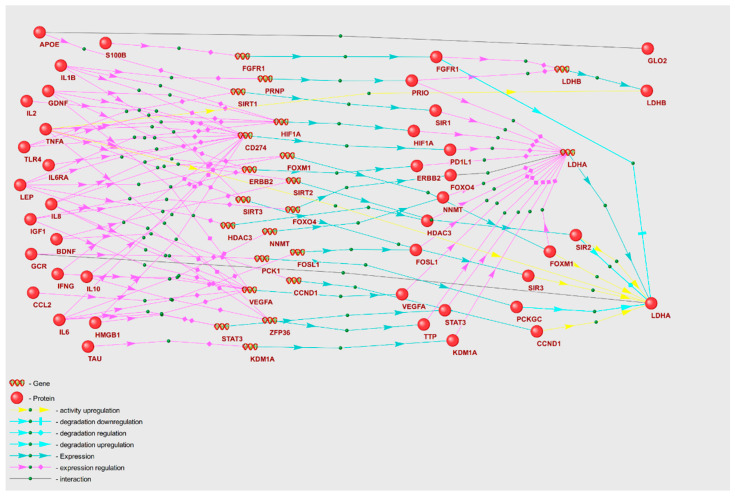
Gene network of regulation of lactate metabolism enzymes by POD genetic markers.

**Figure 6:**
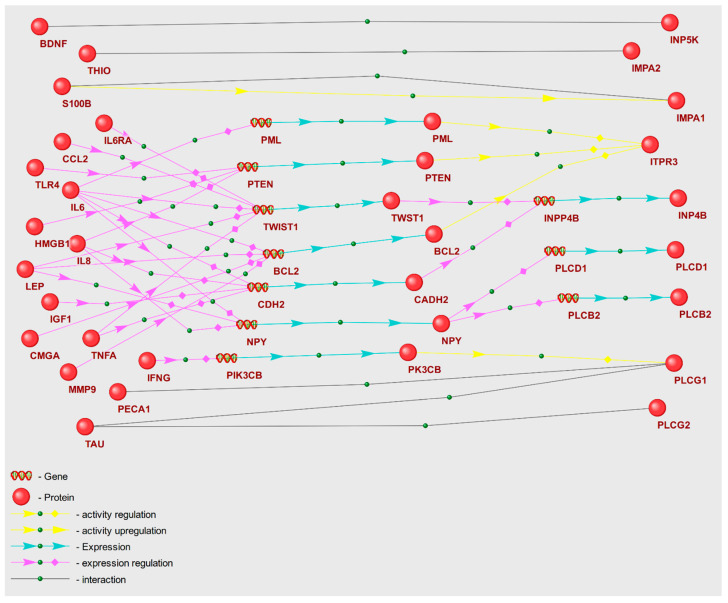
Gene network of regulation of inositol metabolism enzymes by POD genetic markers.

Gene regulatory networks including the enzymes involved in lactate and myo-inositol metabolism are depicted in Figure 5 and Figure 6, respectively. To describe these gene networks, we compiled tables containing POD genetic markers and the enzymes regulated by them, along with the names of KEGG metabolic pathways (Table 7 and Table 8).

As illustrated in Table 7, lactate dehydrogenase chain A (LDHA) is under the most profound regulatory control, with 15 POD genetic markers exerting influence. A total of 19 genetic markers take part in modulation of the enzymes pertinent to lactate metabolic pathways. Concurrently, APOE, IL1B, and TNFA each regulate two corresponding enzymes implicated in lactate metabolism pathways.

As per the data shown in Table 8, the enzymes inositol polyphosphate 4-phosphatase type II (INP4B) and inositol 1,4,5-trisphosphate receptor type 3 (ITPR3) are primarily regulated, with six POD genetic markers directing regulatory links towards them. Fourteen POD genetic markers are implicated in the regulation of enzymes participating in inositol metabolism. Importantly, IL6, IL8, and LEP act as regulators of four enzymes—INP4B, ITPR3, PLCB2, and PLCD1—involved in inositol metabolism pathways.

**Figure 7:**
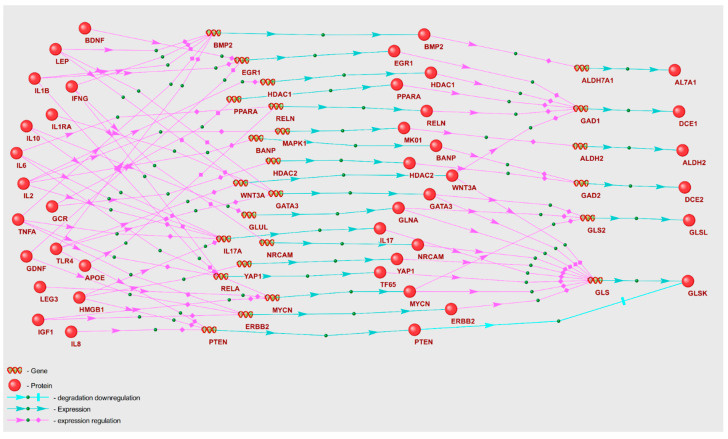
Gene network of regulation of GABA metabolism enzymes by POD genetic markers.

Among the set of metabolites, gamma-aminobutyric acid (GABA) merits specific consideration. Our LS–MS/MS metabolomic analysis revealed a decreased GABA concentration in the POD patients’ group. The gene network delineating the regulation of GABA metabolism enzymes by POD genetic markers is depicted in Figure 7, with principal gene network components shown in Table 9. To reconstruct the gene network, we extracted 13 enzymes implicated in GABA metabolism from the KEGG database.

As shown in Table 9, glutaminase (GLSK) emerges as the primary target of regulatory effects, with 14 POD genetic markers directing their influence towards it. A total of 17 distinct POD genetic markers are implicated in the regulation of GABA metabolism enzymes. Among these are glucocorticoid receptor (GCR) and interleukin 2 (IL2), each modulating four distinct enzymes involved in GABA metabolism.

It is critical to highlight that gamma-aminobutyric acid possesses an inherent inability to cross the blood–brain barrier (BBB). Consequently, any correlation between GABA concentrations in blood plasma and cerebrospinal fluid may be tenuous. To account for the observed relationships between blood plasma and cerebrospinal fluid metabolites, we propose two potential mechanistic explanations. Firstly, we posit that the reconstructed gene regulatory networks may function similarly across various tissue types, including brain cells. Regulatory influences may, thus, drive congruent directional changes in metabolite concentration in cerebrospinal fluid and blood plasma, despite potential disparities in the absolute concentration values. We, therefore, hypothesize that POD genetic markers may induce changes in metabolite concentrations through mechanisms that are analogous in the brain and cerebrospinal fluid. Alternatively, the BBB in POD patients might have experienced localized disruption, thus, fostering partial permeability to metabolites.

**Figure 8:**
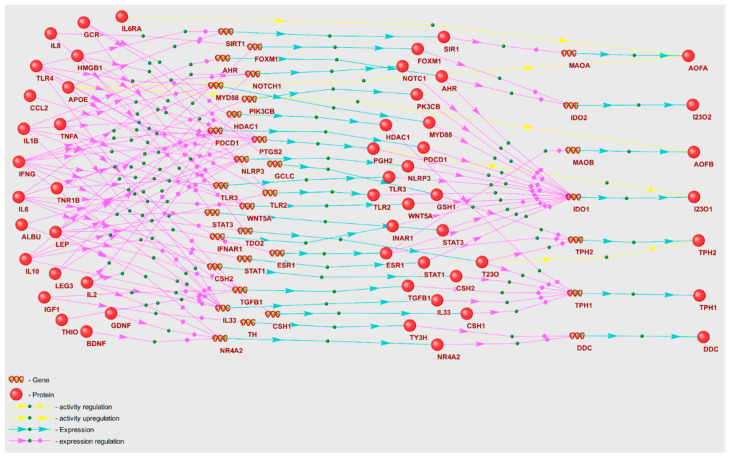
Gene network of regulation of serotonin metabolism enzymes by POD genetic markers.

A noteworthy outcome of our analyses is the identification of serotonin as a secondary metabolomic marker, as outlined in Supplementary Table S5. Given that serotonin was not identified within the overrepresented metabolic pathways, we reconstructed the gene network through which POD genetic markers could potentially modulate serotonin levels. Towards this, we considered nine enzymes derived from two KEGG serotonin metabolism pathways (Figure 8). The key constituents of this gene network are enumerated in Table 10.

In accordance with Table 10, the I23O1 enzyme that is responsible for catalyzing oxidation of L-tryptophan receives the highest number of regulatory impacts from 15 POD genetic markers. A cumulative total of 21 POD genetic markers contribute to the regulation of serotonin metabolism enzymes. Remarkably, leptin (LEP) exerts regulatory control over six enzymes involved in serotonin metabolism—AOFB, DDC, IDO1, IDO2, TPH1, and TPH2.

## 3. Discussion

The understanding of the molecular mechanisms underlying postoperative delirium remains incomplete, despite ongoing research efforts. To date, identification of the prognostic metabolomic markers of POD following major surgical operations remains elusive. This challenge is largely attributed to the complex systemic perturbations at the genetic and metabolomic levels entrenched in POD pathology, which hamper the delineation of discernible patterns using conventional statistical methodologies. In this study, we sought to identify the potential primary and secondary metabolomic biomarkers of POD from metabolomic data. We employed an integrative approach, involving statistical methodologies, the genetic algorithm, and artificial neural networks.

Acknowledging the complex pathophysiological processes inherent in POD, we defined two categories of metabolomic markers to provide a comprehensive description of changes in the metabolomic status of POD patients compared to non-POD patients. Primary markers, within the context of our study, are distinguished by a unidirectional alteration in concentration in blood plasma of patients with POD. Secondary metabolomic markers are typified by disruptions in inter-metabolite correlation relationships.

It is critical to underscore that many of the identified metabolites are incapable of crossing the blood–brain barrier (BBB) from blood plasma to directly influence the central nervous system. Furthermore, the concentrations of such metabolites in blood plasma and cerebrospinal fluid may not correlate. To interpret the results of blood plasma metabolomic analysis that allow us to hypothesize the mechanisms of disturbances in the brain, we postulated two assumptions. Our primary assumption is predicated on the expectation that the reconstructed gene regulatory networks function comparably across diverse tissues. Consequently, it can be anticipated that in various tissues POD genetic markers exert similar effects on metabolite concentration despite potential disparities in the values of metabolite concentrations across different tissues. An alternative hypothesis posits that inflammatory processes in POD could potentiate increased BBB permeability to various metabolites [58,59].

### 3.1. Metabolomic Markers of Postoperative Delirium

Our findings reveal that lactate and inositol are amongst the most prominent potential primary metabolomic markers for postoperative delirium (Supplementary Table S3). Through the use of gene networks, we elucidate the potential regulatory mechanisms regulating the metabolism of lactate and inositol, both of which are significant metabolomic markers of POD (Table 7 and Table 8).

An elevated concentration of lactate detected in patients’ plasma prior to surgery may serve as a risk factor for POD. Lactate plays a crucial role in energy metabolism and can impact tissue perfusion [60]. The concentration of lactate in patients’ plasma during the preoperative and early postoperative periods may function as a prognostic metabolomic marker of POD. Lactic acid, as well as glucose, is vital for providing energy to brain cells. During anaerobic conditions and hypoxia, lactic acid is involved in cellular metabolic pathways and serves as a substrate for tissue perfusion [61]. Lactate has been recognized as a marker of ischemia, hypoxia, and CNS damage [62,63]. The concentrations of glucose and lactate in blood plasma may reflect the stress response to surgical intervention. Decreased levels of glucose and lactate were observed in patients prior to cerebral aneurysm-related surgery [64]. In addition, S-methylcysteine emerged as one of the most important primary metabolomic markers (Supplementary Table S3). Guo et al. identified S-methylcysteine, eicosapentaenoic acid, linolenic acid, and linoleic acid as the metabolomic indicators associated with an elevated risk of POD [65].

Secondary metabolomic markers are characterized by a disrupted correlation with other metabolites, as opposed to a shift towards an increase or decrease in the POD group, as seen with primary markers. Consequently, not all secondary markers demonstrated statistically significant differences in concentration when comparing POD and non-POD groups. Our analysis, intriguingly, identified neurotransmitters as the POD-potential markers. Specifically, serotonin was amongst the secondary metabolomic markers. It was reported that serotonin in the cerebrospinal fluid of the brain is a well-established marker of POD [66,67]. Plasma and CSF serotonin concentrations may not correlate due to the inability of the serotonin molecule to cross the blood–brain barrier. Nonetheless, we propose that disruptions in serotonin metabolism, which identified this neurotransmitter as a secondary marker, may be mediated by the influence of POD genetic markers sharing similar characteristics across various tissues, including brain tissues. Accordingly, the detection of other metabolites that are incapable of crossing the blood–brain barrier may be interpreted as potential markers of POD. Another example of a neurotransmitter marker is GABA, which was identified as both a primary and secondary metabolomic marker of POD.

### 3.2. Overrepresented Metabolic Pathways

Through the analysis of KEGG metabolic pathways, overrepresented within both the primary and secondary markers, a set of statistically significant metabolic pathways were discerned (Table 3 and Table 4). Notably, the analysis conducted exclusively on the primary marker set did not yield statistically significant results, likely due to the intricate nature of metabolic pathway dysfunction. This complexity encompasses not only directional shifts in metabolite concentrations but also the coordination in the variability of metabolite pathway participants. Consequently, the need for advancing bioinformatic methods for identifying secondary metabolomic markers is underscored.

The overrepresented metabolic pathways identified herein are frequently related to amino acid metabolism. It is established that mechanisms such as amino acid metabolism pathway disorders, fatty acids, and the activation of alternative energy metabolic processes are implicated in the pathogenesis of POD [66,68]. Guo et al., in their study of patients who underwent arthroplasty following a hip fracture, identified oxidative stress disorders, disruptions in energy metabolism, and amino acid metabolism pathways in patients with POD [69]. According to the report, alterations in fatty acid and amino acid metabolism could take part in the pathophysiology of POD.

Tripp et al. conducted a computational analysis of blood plasma metabolomic profiles in elderly surgical patients. Based on metabolomic data, they conducted an overrepresentation analysis of KEGG metabolic pathways, which revealed alterations in the “Valine, leucine, and isoleucine biosynthesis” pathway in patients with POD on the day prior to surgery, and metabolites of the “Citrate cycle” pathway on the second day postoperatively [70]. Interestingly, we also identified the “Valine, leucine, and isoleucine biosynthesis” pathway as overrepresented within the primary and secondary metabolomic markers’ set.

Takahashi et al. constructed a protein–protein interaction network and identified two sets of human genes associated with POD development [71]. Their overrepresentation analysis revealed KEGG metabolic pathways enriched with POD genetic markers, including “neuroactive ligand-receptor interaction”, cAMP, IL17, and TNF signaling pathways. Among the Gene Ontology terms enriched with POD genetic markers were “glutamate receptor activity”, “neurotransmitter receptor activity”, and “G protein-coupled serotonin receptor activity”. In line with our findings, the metabolic pathways including neurotransmitter metabolism were among the overrepresented metabolic pathways.

### 3.3. Gene Networks Regulating Metabolic Pathways with POD Genetic Markers

Recently, several genetic markers for postoperative delirium, implicated extensively in the pathogenesis of POD, have been identified [72]. These markers are supposed to take part in initiating molecular pathways linked to the pathophysiology of delirium, including neuroinflammation, blood–brain barrier disruption, neurotransmitter imbalance, oxidative stress, neuroendocrine dysregulation, and neuronal connectivity disorganization [73]. By reconstructing and analyzing gene networks, we propose that these genetic markers, when dysregulated in the POD group, can instigate systemic disturbances in metabolic pathways. This is achieved through abnormal regulation of enzyme expression, activity, and stability (Figure 2 and Figure 3). Among the analyzed POD genetic markers (Supplementary Table S6), TNFA is involved in regulation of the greatest number of enzymes within the overrepresented metabolic pathways (Figure 4). Consequently, these genetic markers may serve as the key factors in metabolic pathway dysfunction identified via overrepresentation analysis.

As illustrated in the gene network graph (Figure 3), interleukin-6 could potentially regulate SYAC and SYAM enzymes of the “Aminoacyl-tRNA biosynthesis” metabolic pathway, with the involvement of granzyme B and FTO protein. In the presence of IL6 and inflammation, *GZMB* gene expression may be decreased [74,75]. Granzyme B enzyme can cleave histidyl-, isoleucyl-, and alanyl-tRNA synthetases [76]. Concurrently, via the STAT3 signaling pathway, IL6 is capable of enhancing FTO gene expression. Notably, FTO deficiency can result in diminished expression of LRS (leucyl-tRNA synthetase, SYLC) and other aminoacyl-tRNA synthetase family enzymes [77]. These enzymes catalyze the binding of amino acids to aminoacyl-tRNA, thereby directly influencing the primary metabolomic markers identified as alanine, leucine, and isoleucine.

Although our metabolomic analysis of blood plasma revealed these amino acids to be decreased in the POD group (Supplementary Table S1), this reduction aligns with the anticipated regulatory effect of *IL6* overexpression, as suggested by the gene network. Preoperative elevation of IL6 was observed in POD patients [78]. Another regulatory pathway example is the IFNG→Tryptophanyl-tRNA synthetase pathway (Figure 3). As per the gene network, IFNG directly enhances *WARS1* (Tryptophanyl-tRNA ligase 1) expression, and that upregulation may result in a decrease in tryptophan content [79]. Our data identified tryptophan as a primary metabolomic marker, and this metabolite was found to be decreased in the POD group. It is crucial to consider the whole set of regulatory effects on the target gene described by the gene network when assessing the impact of a specific regulatory pathway. The interference of multiple regulatory pathways, which together form the gene network, necessitates the use of mathematical modeling methods. Therefore, in future studies, we are planning to interpret regulatory pathways at a qualitative level.

In an investigation of the regulatory pathways that potentially disrupt the function of enzymes in the “Glycine, serine, and threonine metabolism” pathway, IFNG→BHMT1, IL1B→BHMT1, and TNFA→BHMT1 emerge as important contributors (Figure 2). The gene network suggests that IFNG, IL1B, and TNFA can stimulate *RELA* gene expression, which encodes the TF65 transcription factor. TF65 in turn can inhibit the transcriptional activity of the *BHMT1* (Betaine-homocysteine S-methyltransferase 1) promoter. Of particular note, the BHMT1-mediated conversion involves secondary metabolomic markers L-homocysteine and L-methionine [80].

Investigating the potential influence of POD genetic markers on the deviant regulation of metabolomic markers that were not incorporated into the primary metabolic profile (PMP), we reconstructed gene networks for metabolic pathways housing the relevant enzymes. This enabled us to postulate regarding the principal regulators of these relevant enzymes within the set of POD genetic markers. As per our prior demonstration (Table 7), the regulation of inositol was primarily influenced by IL6, IL8, and LEP, while lactate regulation was mainly driven by APOE, IL1B, and TNFA.

For instance, the gene network in Figure 5 indicates that IL6 regulates the expression of *CD274*, *FOSL*, *NNMT*, *STAT3*, and *VEGFA*, whose protein products subsequently regulate the expression of *LDHA* (L-lactate dehydrogenase A chain). LDHA mediates the reversible conversion of lactate to pyruvate. It is noteworthy that associations between *STAT3* and *VEGFA’s* elevated expression and POD development were identified in prior studies [57,81]. The enzyme NNMT catalyzes the transmethylation of nicotinamide. Intriguingly, the *FOSL* (Fos-related antigen 1) and *CD274* (Programmed cell death 1 ligand 1) genes are linked with apoptosis. It was established that the apoptotic processes in brain neurons constitute a part of the pathophysiological manifestations of postoperative delirium [82].

From the gene networks of GABA and serotonin regulation by genetic markers (Figure 7 and Figure 8), it is evident that serotonin is subject to more regulatory influence than GABA. A unique attribute of the serotonin gene network is the multifaceted regulation of the I23O1 enzyme, which catalyzes the oxidation reaction of L-tryptophan. This enzyme was reported to be associated with POD due to its contribution to a decrease in L-tryptophan levels and a concurrent increase in kynurenine levels [83]. Notably, our analytical results identified L-tryptophan and kynurenine as the potential POD metabolomic markers (Supplementary Tables S3 and S5).

In the analysis of pathologies such as postoperative delirium using blood plasma metabolomic data, a pertinent question arises regarding the extent to which alterations in the plasma metabolomic profile can represent changes in the cerebrospinal fluid (CSF) and metabolomic status of the brain. Studies investigating correlations between changes in blood plasma and CSF metabolomic profiles are, therefore, of considerable relevance [21]. In this study, we hypothesize that certain metabolic pathways linked to POD development are disrupted in both blood plasma and CSF. This could particularly be evidenced by detection of elevated oxidative stress in cerebrospinal fluid, which can significantly impact the functioning of the nervous system. Preoperative low CSF saturation was previously suggested as being a potential prognostic marker of POD [84]. In another study, based on CSF metabolomic analysis in patients undergoing arthroplasty, Pan et al. identified spermidine, putrescine, and glutamine as potential prognostic markers of POD [85]. The authors proposed the ratio of spermidine and putrescine concentrations to beta-amyloid 42 (Aβ42) as a potential marker of postoperative delirium.

Notably, changes in the content and correlation of metabolites in blood plasma can reveal disruptions in the pathways of amino acid biosynthesis, energy metabolism, and oxidative stress. These disturbances can, in turn, contribute to the pathophysiology of POD [69]. According to the reconstructed gene networks, disruptions in the functioning of metabolic pathways leading to alterations in metabolomic profiles in the POD group can primarily be associated with the modified functions of POD genetic markers.

The molecular mechanisms of postoperative delirium remain incompletely resolved, and the task of delineating reliable prognostic metabolomic markers post various surgical operations continues to present a formidable challenge—most predominantly in cardiac surgery. The complex nature of metabolomic data analysis in postoperative delirium studies can be attributed to the multifaceted systemic perturbations at the genetic and metabolomic levels inherent in this pathology, consequently impeding the attainment of robust results via conventional statistical methodologies.

In this study, we put forward several innovative computational strategies for discerning potential primary and secondary metabolic markers of POD that were grounded in the examination of blood plasma metabolomic data from patients. These strategies encompass the deployment of the genetic algorithm coupled with artificial neural networks. Employing these methodologies to analyze blood plasma metabolomic data from patients, harvested after cardiac surgery, facilitated the identification of a constellation of primary and secondary potential metabolomic markers of POD. L-Lactic acid, inositol, methylcysteine, and Adenine emerged as the pivotal primary markers that were indicative of the metabolic concentration shifts in the POD group. A set of 54 metabolites, inclusive of neurotransmitters such as GABA and serotonin, were categorized as the secondary markers, and were characterized by their disrupted correlations with other metabolites in the POD group.

Analysis of metabolic pathway overrepresentation, along with the reconstruction of gene regulatory networks, afforded us the opportunity to postulate about the interplay between perturbations in metabolic pathway functionality associated with a predisposition to POD and established genetic markers of POD, such as TNFA, LEP, IL6, and others. It is plausible to conjecture that functional disruptions in genetic POD markers could have an expansive influence on a spectrum of pathophysiological mechanisms, and that these genes have the potential to impact the metabolomic profile of patients. Collectively, the findings underscore the systemic essence of metabolic disturbances within patients exhibiting POD.

Further experimental validation of the proposed hypotheses concerning the role of POD genetic markers in the aberrant regulation of metabolic pathways could potentially emerge as a promising trajectory in the exploration of POD pathogenesis. The suggested approach of bioinformatic analysis of metabolomic data utilizing the genetic algorithm and artificial neural network methodologies holds possibilities for the identification of primary and secondary potential markers across a wide amount of metabolomic studies.

### 3.4. Study Limitations

While our study provides valuable insights into the metabolomic markers of postoperative delirium (POD), it is important to acknowledge several limitations that may affect the generalizability and interpretation of our findings.

Firstly, the study was conducted with a relatively small sample size. We did not perform a priori calculations to determine the necessary sample size for achieving adequate statistical power. This limitation may have affected the robustness of our results and potentially led to overestimation or underestimation of certain effects.

Secondly, our study focused exclusively on patients undergoing cardiac surgery. While this allowed for a more controlled environment and reduced variability due to different surgical procedures, it also limits the applicability of our findings to other surgical populations. POD can occur across various surgical specialties, and the metabolomic profile associated with its development may differ in non-cardiac surgeries.

Thirdly, the homogeneity of our patient population restricts the generalizability of our results. Our findings need to be confirmed in a larger and more heterogeneous patient cohort, including individuals from different age groups, ethnicities, and with varying comorbidities.

Lastly, while our AI-based approach yielded promising results, it is important to note that these findings are primarily exploratory. Further validation studies are necessary to confirm the clinical utility and predictive power of the identified metabolomic markers.

Despite these limitations, our study provides a foundation for future research in this area and highlights the potential of AI-assisted metabolomic analysis in understanding and predicting POD. Addressing these limitations in future studies will be crucial for advancing our knowledge and developing more robust predictive models for POD.

## 4. Materials and Methods

### 4.1. Study Subjects

This study included patients aged 65 and above who underwent cardiac surgery involving cardiopulmonary bypass (CPB). Exclusion criteria included emergency intervention, aortic surgery, significant carotid artery stenosis, Parkinson’s disease, liver cirrhosis (Child–Pugh class B or C), and the consumption of anticholinergic drugs, antidepressants, antiepileptic medications, and chemotherapy drugs. Patient recruitment transpired between June 2019 and January 2021, resulting in inclusion of 39 patients in the study. Table 11 presents biometric data of the participants. Within five days post-surgery, the presence of postoperative delirium was ascertained using the CAM-ICU test (Confusion Assessment Method for the Intensive Care Unit). The initial test was conducted 6–8 h post-surgery, followed by twice daily assessments. A positive CAM-ICU test at any time point was indicative of postoperative delirium.

To provide a clearer picture of our study population, we have compiled the baseline demographic, clinical, surgical, and postoperative characteristics of patients undergoing cardiac surgery in Table S7. This comprehensive dataset offers valuable insights into the diverse factors that may influence our research outcomes. In addition to the patient characteristics, we have organized all the key elements of our study design into Table S8. This table presents a concise yet thorough overview of our research protocol, detailing the inclusion and exclusion criteria, the process for selecting participants, our methods for data collection, and the analytical approaches we employed.

### 4.2. Blood Sampling and Preparation

Venous blood samples were collected from patients 24 h after cardiac surgery, with a 9 mL BD Vacutainer^®^ KEDTA tube containing potassium EDTA as an anticoagulant. Plasma was obtained via centrifugation at 2000× *g* and 4 °C for a duration of 15 min, then portioned and cryopreserved at −80 °C until its deployment for analysis.

Adhering to the protocol proffered by Li et al. [86], all plasma samples were processed in aggregate. For each, 100 μL aliquots of blood plasma was mixed with 400 μL cooled methanol/acetonitrile solution (1:1 ratio), vigorously shaken, and subsequently centrifuged at 16,000 rpm and +4 °C for 15 min. The supernatant was transferred into a glass vial insert for subsequent analysis. In parallel, two quality control samples were prepared by mixing equal volumes of blood plasma derived from the groups with and without POD.

### 4.3. LC–MS/MS Analysis

HPLC–MS/MS analysis was conducted using a Shimadzu LC-20AD Prominence chromatograph, which featured a binary gradient pump, a cooling autosampler SIL-20AC thermostated at 10 °C, and a CTO-10ASvp column oven maintained at 40 °C. A reversed-phase column octadecylsilyl sorbent ProntoSIL 120-5 C18 AQUA (LLC Econova, Novosibirsk, Russia), was employed for the LC separation. The mobile phase A consisted of water with 0.1% HCOOH (*v*/*v*), while mobile phase B was pure acetonitrile. The elution gradient proceeded is as follows: from 0 to 2 min, the B content was 3%; from 2 to 7 min, it increased to 10%; from 7 to 10 min, it reached 90%; and from 10 to 12 min, it was increased to 100% and was maintained until 16.5 min. After the run, the column was equilibrated for 3.4 min with 3% B. The flow rate was 200 μL/min, and a 2 μL sample injection volume was used.

For mass selective detection, a 6500 QTRAP mass spectrometer (AB SCIEX, Framingham, MA, USA), which was equipped with an electrospray ionization source operating in both positive and negative modes, was utilized. The mass spectrometer was set to operate in multiple reaction monitoring (MRM) mode for metabolite detection with the following parameters: the ion spray voltages (IS) were 5500 V for positive mode and −4500 V for negative mode. The gas dryer temperature (TEM) was set at 475 °C, collision-induced dissociation gas (CAD) was configured as “Medium”, and the sprayer gas (GS1), dryer gas (GS2), and curtain gas (CUR) were set to 35, 35, and 30 psi, respectively. The declustering potential (DP) was ±93 V, the entrance potential (EP) was ±10 V, and the collision cell exit potential (CXP) was ±20 V for positive and negative ion modes, respectively. Additionally, the time for polarity switching (settling time) was established at 5 ms, and the dwell time was 3 ms for each MRM transition. The precursor ion and fragment ion transitions, metabolite names, dwell times, and the appropriate collision energies for both positive and negative ion modes were adapted from Yuan et al. [87], with several metabolite transitions added by our research group. The instrument was controlled, data were collected using Analyst 1.6.2 software (AB SCIEX), and chromatograms were processed using MultiQuant 2.1 software (AB SCIEX). The obtained peak area values were used for the following statistical analysis.

### 4.4. Data Preprocessing

The raw data were preprocessed to fill in missing values for metabolite content within the analyzed samples. In instances where the number of samples with missing values did not exceed 5% of the total value number across 39 patients, the median value derived from remaining samples was substituted as the metabolite content value. This approach is validated by robustness of the median to outliers. The ensuing values were further log-transformed.

### 4.5. Data Analysis

**Figure 9:**
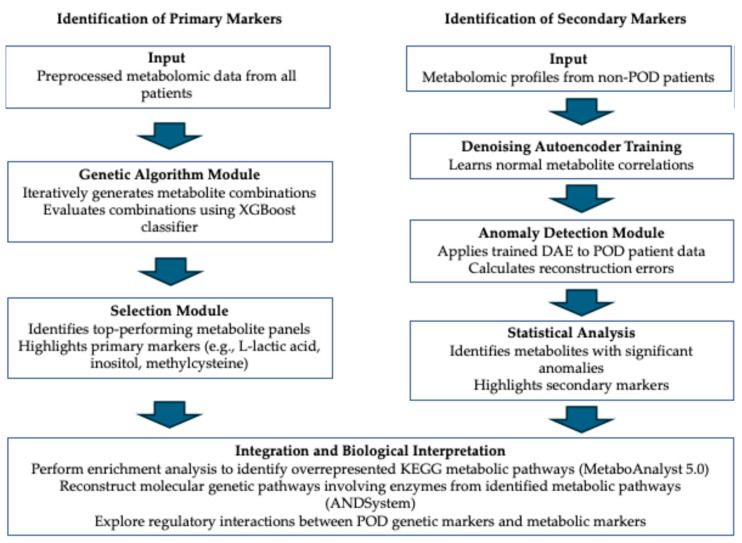
Schematic workflow for identification of primary and secondary metabolomic markers.

Our data analysis approach for identifying and validating metabolomic markers associated with postoperative delirium utilized a comprehensive set of computational methods. Figure 9 provides a schematic workflow that visually represents our analytical process, highlighting the computational algorithms and methodologies employed. To identify primary markers that best distinguish between POD and non-POD patients, we conducted an initial statistical analysis, followed by the application of a genetic algorithm. To search for secondary markers, we trained a denoising autoencoder (DAE) to detect anomalies in POD patients. This step allowed us to identify metabolites with significantly higher reconstruction errors in POD patients, indicating disrupted metabolic correlations. By combining primary and secondary markers, we gained a more comprehensive understanding of the metabolic alterations occurring in POD. To explore the biological significance of the identified markers, we performed metabolic pathway and gene network analyses.

#### 4.5.1. Statistical Analysis

The significance of difference between mean metabolite levels in POD and non-POD patients was computed using the Mann–Whitney test, implemented in the SciPy package v1.8.0 (https://scipy.org/, accessed on 10 November 2022). The multiple hypothesis testing was enacted using the Benjamini-Yekutieli procedure from the statsmodels Python package v0.13.231 (https://www.statsmodels.org/stable/index.html, accessed on 10 November 2022).

#### 4.5.2. Genetic Algorithm

To identify the combinations of metabolites that differ most effectively between POD and non-POD patient groups, we employed the genetic algorithm, as described by Lu et al., 2017 [54]. This algorithm was executed through the application of a Python module PyGAD v3.2.0 (https://pygad.readthedocs.io, accessed on 17 November 2023). The fitness function was formulated in the following manner: (1) an XGBoost classification model v2.1.2 (https://xgboost.readthedocs.io/, accessed on 17 November 2023) was constructed utilizing the input list of metabolites and (2) the precision of this model was evaluated by a 5-fold cross-validation methodology, with selection of the minimum accuracy value. The function returned the calculated model precision. The XGBoost model was trained with the following parameters: n_estimators = 1000, learning_rate = 0.05, and other parameters set to their default values.

#### 4.5.3. Denoising Autoencoder (DAE)

The DAE architecture comprised three fully interconnected layers (input, hidden, and output), each utilizing the Rectified Linear Unit (ReLU) activation function. The input and output layers contained 210 neurons, which corresponds to the number of metabolites analyzed. The hidden layer, depending upon the specific list, contained from 50 to 149 neurons. The optimizer applied was Adam, with a learning rate of 0.001. The model was trained over 20 epochs, with a batch size of 8 and Mean Squared Error (MSE) as the loss metric. Data were split into training (80%) and testing (20%) sets. The DAE model was implemented using the PyTorch package v2.2.1 (https://pytorch.org/, accessed on 17 November 2023). During the training phase, the DAE input contained metabolomic profile with noise added to the metabolite concentration values. The DAE aimed to encode the input data and predict the original data prior to addition of noise.

#### 4.5.4. Generation of Noisy Metabolomic Profile

A noisy metabolomic profile was generated by introducing a random number, derived from a normal distribution to each metabolite concentration value. The formula applied was as follows:

*CN*_*n*_ = *CO_n_* + *d*·*e*,

where *CN_n_* denotes the noisy concentration value of the *n*-th metabolite, *CO_n_* represents the original concentration value of the *n*-th metabolite, *e* is a member of a normal distribution with the parameters *N* (*Ex* = 0, *σ* = *CO_n_*), and *d* is the noise level factor.

The coefficient *d* was assigned a value of 0.25 for distinct DAE models. This procedure permitted the generation of 500 noisy profiles from a single original metabolomic profile.

#### 4.5.5. Identification of the Secondary Metabolomic Markers

To explore secondary metabolomic markers, we adopted a technique for anomaly detection by employing a denoising autoencoder, as delineated by Sakurada et al., 2014 [49]. The algorithm comprised the following steps: initially, the denoising autoencoder was trained using a set of metabolomic profiles from patients without postoperative delirium (non-POD group). Subsequently, the trained model was utilized to examine the metabolomic profiles pertaining to the POD patients’ group. Metabolites manifesting the anomalous concentration values, as per the denoising autoencoder, were classified as the secondary metabolic markers.

To evaluate the anomaly degree of each metabolite in the POD patient group, we contrasted the distribution of denoising autoencoder errors when analyzing the POD and non-POD groups. The error was gauged as the absolute value of the discrepancy between the metabolite concentration values supplied to the denoising autoencoder input and those procured at the output. Crucially, the original metabolomic profiles, devoid of noise, were supplied to the denoising autoencoder input. Disparities between error distributions were assessed via the Mann–Whitney test. Data were deemed to contain anomalies if the two distributions exhibited statistically significant differences. Multiple hypothesis testing was enacted using the False Discovery Rate (FDR) Benjamini–Hochberg procedure.

The architecture of the denoising autoencoder involves the hidden layer with a lower dimensionality than either the input or output layers. It can be hypothesized that the manifestation of anomalies in the metabolite concentration data, as identified via the denoising autoencoder, is associated with a disruption of relationships (correlation links) between metabolites in POD patients. Such disruptions could be linked to dysfunctions in metabolic processes, engendering a predisposition to POD.

### 4.6. MetaboAnalyst Enrichment Analysis

The analysis of overrepresented KEGG metabolic pathways was evaluated using the enrichment analysis instrument implemented in the MetaboAnalyst 5.0 web tool [41] (https://www.metaboanalyst.ca/, accessed on 10 November 2022).

### 4.7. Reconstruction of Gene Networks

Gene networks’ reconstruction was carried out using ANDVisio program, which serves as a graphical user interface for the ANDSystem cognitive system. Using the “Pathway Wizard” module in the ANDVisio program, we reconstructed molecular genetic pathways of regulation of identified metabolic pathways enzymes by POD genetic markers. Regulatory pathways were built according to five template types, presented in Table 12. These templates include various combinations of molecular genetic interactions, such as regulation of gene expression, protein–protein interactions, regulation of protein activity, degradation, catalysis, and transport.

## 5. Conclusions

In this study, we introduced a comprehensive computational approach utilizing artificial intelligence methods to identify potential primary and secondary metabolomic markers of postoperative delirium (POD). By analyzing the preoperative blood plasma metabolomic profiles of patients undergoing cardiac surgery, we were able to discern combinations of metabolites that effectively distinguish between patients who developed POD and those who did not. We selected patients who underwent cardiac surgery because such interventions are associated with a high risk of developing postoperative delirium due to the specifics of open-heart surgery, the use of cardiopulmonary bypass, and the impact on the central nervous system. Our findings represent one of the first applications of such AI methodologies in the context of POD, marking a significant advancement in the field of metabolomics and its intersection with artificial intelligence.

The primary metabolomic markers identified, including L-lactic acid, inositol, and methylcysteine, demonstrated a strong predictive capability for POD manifestation, achieving an area under the ROC curve (AUC) of 99%. These markers indicate specific shifts in the metabolite concentrations associated with POD. Importantly, we also identified a set of 54 secondary metabolomic markers characterized by disrupted correlations with other metabolites in the POD group. Among these secondary markers were neurotransmitters such as gamma-aminobutyric acid (GABA) and serotonin, suggesting systemic disturbances in neurotransmitter metabolism that are not evident through concentration changes alone.

Our approach underscores the importance of considering both primary and secondary metabolomic markers to gain a comprehensive understanding of systemic metabolic disruptions in pathological conditions like POD. Traditionally, such secondary markers remain unexplored in metabolomic studies due to the complexity of their detection and interpretation. By employing a denoising autoencoder to detect anomalies in metabolite correlations, we highlighted the significance of inter-metabolite relationships and their perturbations in POD patients.

Our study contributes a novel analytical framework that can be extended beyond POD to other pathologies characterized by complex metabolic alterations. The combination of genetic algorithms and neural networks for metabolomic data analysis enhances the ability to detect subtle but clinically significant biomarkers, which could lead to more accurate predictions of disease development and progression.

For future research, it is imperative to validate the proposed markers experimentally in larger, independent cohorts to confirm their predictive power and clinical utility. Additionally, exploring the integration of genomic, proteomic, and metabolomic data using AI methodologies may further elucidate the molecular mechanisms underlying POD and other complex disorders. Such integrative analyses could facilitate the development of personalized medicine approaches, including preventive strategies and tailored therapeutic interventions.

In conclusion, our study not only advances the understanding of POD pathogenesis by highlighting systemic metabolic disturbances but also introduces a robust AI-assisted methodology for metabolomic analysis. This work paves the way for future explorations into the systemic nature of metabolic dysfunctions in various diseases and underscores the potential of AI in uncovering clinically relevant biomarkers.

## Figures and Tables

**Table 1:** Analysis of overrepresented KEGG metabolic pathways associated with the set of primary metabolomic markers.

KEGG Pathway	Total	Metabolomic Markers (Hits)	*p*-Value	FDR
Pyrimidine metabolism	39	3	0.006	0.256
Tryptophan metabolism	41	3	0.006	0.256
Tryptophan metabolism	41	3	0.028	0.616

**Table 2:** Overrepresented KEGG metabolic pathways for the set of secondary markers identified by 100 autoencoder models.

KEGG Metabolic Pathway	Total	Hits	*p*-Value	FDR
beta-Alanine metabolism	21	2	0.006	0.403
Glycine, serine and threonine metabolism	33	3	0.015	0.403

**Table 3:** Overrepresented KEGG metabolic pathways for the set of secondary markers identified by 97 autoencoder models.

KEGG Metabolic Pathway	Total	Hits	*p*-Value	FDR
Glycine, serine, and threonine metabolism	33	4	2.03 × 10^−4^	0.0163
beta-Alanine metabolism	21	3	8.99 × 10^−4^	0.036

**Table 4:** Metabolites serving as both the primary and secondary metabolomic markers.

KEGG ID	Metabolite	Primary Marker Importance
C00137	Inositol	87
C00120	Biotin	3
C01089	3-hydroxybutyric acid	1
C00318	Carnitine	1
C00334	Gamma-Aminobutyric acid	1
C00258	Glyceric acid	1
C00106	Uracil	1
C00438	Ureidosuccunic acid	1

**Table 5:** Overrepresented KEGG metabolic pathways for the combined set of primary and secondary metabolomic markers. Metabolites that are primary and secondary markers are marked in bold.

KEGG Pathway	Total	FDR	Primary Metabolomic Markers (Hits)	Secondary Metabolomic Markers (Hits)
Glycine, serine, and threonine metabolism	33	0.0021	Betaine; **Glyceric acid**	L-Serine; Choline; Betaine aldehyde; Guanidoacetic acid; Cystathionine; L-Threonine; **Glyceric acid**
Aminoacyl-tRNA biosynthesis	48	0.0028	L-Leucine; L-Tryptophan; L-Tyrosine	L-Histidine; L-Arginine; L-Serine; L-Valine; L-Threonine; L-Proline
Tryptophan metabolism	41	0.0239	L-Tryptophan; 5-Hydroxy-L-tryptophan	Serotonin; 3-Hydroxyanthranilic acid; L-Kynurenine; Acetyl-CoA
Arginine biosynthesis	14	0.0351	N-Acetylglutamic acid	L-Arginine; Citrulline; Ornithine
Butanoate metabolism	15	0.0372	**3-hydroxybutyric acid; Gamma-Aminobutyric acid**; D-2-Hydroxyglutaric acid	**3-hydroxybutyric acid**; Acetyl-CoA; **Gamma-Aminobutyric acid**
Valine, leucine, and isoleucine biosynthesis	8	0.0375	L-Leucine	L-Threonine; L-Valine
Arginine and proline metabolism	38	0.0375	**Gamma-Aminobutyric acid**	L-Arginine; Guanidoacetic acid; **Gamma-Aminobutyric acid**; Spermine; Ornithine; L-Proline
Pyrimidine metabolism	39	0.0376	Cytidine; Thymine; **Ureidosuccinic acid**; **Uracil**	Uridine; **Ureidosuccinic acid**; **Uracil**; Deoxyribose 1-phosphate
Ubiquinone and other terpenoid-quinone biosynthesis	9	0.0394	L-Tyrosine; Homogentisic acid	Phenyllactic acid

**Table 6:** Quantitative indicators of POD genetic markers (N1) and their corresponding regulated metabolic pathway enzymes (N2), as per regulatory pathway templates.

KEGG Pathway	Regulatory Pathways									
	P1		P2		P3		P4		P5	
	N1	N2	N1	N2	N1	N2	N1	N2	N1	N2
Glycine, serine, and threonine metabolism	2	3	3	5	3	3	15	13	5	3
Aminoacyl-tRNA biosynthesis	1	1	0	0	1	2	10	3	5	2

**Table 7:** Descriptive features of gene network of regulation of lactate metabolic pathway enzymes.

№	KEGG Lactate Metabolism Pathways	Enzymes in the Metabolic Pathway	Regulatory Genetic Markers
1	Glycolysis/Gluconeogenesis: hsa00010;	LDHA	APOE, CCL2, GDNF, HMGB1, IGF1, IFNG, IL1B, IL2, IL6, IL6RA, IL8, IL10, LEP, TLR4, TNFA
		LDHB	IL1B, S100B, TNFA
2	HIF-1 signaling pathway: hsa04066	LDHB	IL1B, S100B, TNFA
		LDHC, LDHAL6A, LDHAL6B	-
3	Pyruvate metabolism: hsa00620	GLO2	APOE
		LDHA	APOE, CCL2, GDNF, HMGB1, IGF1, IFNG, IL1B, IL2, IL6, IL6RA, IL8, IL10, LEP, TLR4, TNFA
		LDHB	IL1B, S100B, TNFA
		GLUL, GRHPR, HAGH, LDHC, LDHD, LDHAL6A, LDHAL6B	-

**Table 8:** Characteristics of gene regulatory network of inositol metabolic pathway enzymes.

№	KEGG inositol Metabolism Pathways	Enzymes in the Metabolic Pathway	Regulatory Genetic Markers
1	Inositol phosphate metabolism: hsa00562	IMPA1	S100B
		IMPA2	THIO
		INP4B	CCL2, IGF1, IL6, IL6RA, IL8, LEP
		INP5K	BDNF
		ITPR3	HMGB1, IL6, IL8, LEP, MMP9, TLR4
		CDIPT, INP1, INP4A, ISYNA1, MIOX, MTM1	-
2	Phosphatidylinositol signaling system: hsa04070	IMPA1	S100B
		IMPA2	THIO
		INP4B	CCL2, IGF1, IL6, IL6RA, IL8, LEP
		PLCB2, PLCD1	IL6, IL8, LEP
		PLCG1	PECA1
		PLCG2	TAU
		BPNT2, CDIPT, INP1, INP4A, INP5A, MTM1, PLCs	-
3	Ascorbate and aldarate metabolism: hsa00053	MIOX	-

**Table 9:** Characteristics of gene regulatory network of GABA metabolic pathway enzymes.

№	KEGG GABA Metabolism Pathways	Enzymes in the Metabolic Pathway	Regulatory Genetic Markers
1	GABAergic synapse: hsa04727	GLSK	GDNF, GCR, HMGB1, IGF1, IFNG, IL1B, IL1RA, IL2, IL6, IL8, IL10, LEG3, LEP, TLR4
		GLSL	GCR, IGF1, IL1RA, IL6, IL8, IL10, LEG3, LEP, TLR4
		GAD1	APOE, BDNF, GCR, GDNF, IL1B, IL2, IL6
		GAD2	GCR, IL2
		GABT	-
2	Arginine and proline metabolism: hsa00330	ALDH2	IL2
		ALDH1B1, ALDH3A2, ALDH7A1, ALDH9A1, CNDP1, CNDP2, GATM	-
3	Butanoate metabolism: hsa00650	GAD1	APOE, BDNF, GCR, GDNF, IL1B, IL2, IL6
		GAD2	GCR, IL2
		GABT	-

**Table 10:** Characteristics of gene regulatory network of the enzymes in serotonin metabolic pathways.

№	KEGG Serotonin Metabolism Pathways	Enzymes in the Metabolic Pathway	Regulatory Genetic Markers
1	Serotonergic synapse: hsa04726	TPH1	ALBU, GCR, IGF1, IL1B, IL2, IL6, IL10, LEP, THIO, TLR4, TNFA
		DDC	BDNF, GDNF, IGF1, IL2, LEP
		TPH2	GCR, LEP
		AOFA	APOE, TLR4
		AOFB	APOE, LEP
2	Tryptophan metabolism: hsa00380	I23O1	ALBU, CCL2, GCR, HMGB1, IFNG, IL1B, IL2, IL6, IL8, IL10, LEG3, LEP, TLR4, TNFA, TNR1B
		DDC	BDNF, GDNF, IGF1, IL2, LEP
		I23O2	IL2, LEP
		AOFA	APOE, TLR4
		AOFB	APOE, LEP
		INMT, SNAT	-

**Table 11:** Age and gender characteristics of the patients.

Group	Sex (M/F)	Min. Age	Max. Age	Avg. Age	Median	Standard Deviation
Non-POD	11/16	65	75	69.6	70	3.0
POD	5/7	65	79	69.7	69.5	4.3

**Table 12:** Templates for molecular genetic pathways of metabolic pathway enzymes regulation by POD genetic markers.

№	Template Name	Regulatory Pathway
P1	Protein–protein interactions	PODp *–protein–protein interaction→EnzP
P2	Protein function regulation	PODp–regulation of activity/degradation/catalyze/transport→EnzP
P3	Expression regulation	PODp–expression regulation→EnzG–expression→EnzP
P4	Double expression regulation	PODp–expression regulation→Hg–expression→Hp –expression regulation→EnzG–expression→EnzP
P5	Combined regulatory pathway	PODp–expression regulation→Hg–expression→Hp–regulation of activity/degradation/catalyze/transport→EnzP

* Abbreviations: PODp—POD genetic markers proteins; Hg—human genes; Hp—human proteins; EnzG—genes encoding metabolic pathway enzymes; and EnzP—metabolic pathway enzymes.

## Data Availability

The data are provided with this paper in the Supplementary Information. The source code of AIMetabolicMarker and used data are available at https://github.com/LCP-ICG/AIMetabolicMarker, accessed on 3 September 2024.

## Supplementary Materials

The following supporting information can be downloaded at: https://www.mdpi.com/article/10.3390/ijms252111847/s1.
