# Supplementary material for: AI-Assisted Identification of Primary and Secondary Metabolomic Markers for Postoperative Delirium
Source: Int J Mol Sci. 2024 Nov 4;25(21):11847. doi: 10.3390/ijms252111847 (PMC11546914; doi:10.3390/ijms252111847)
Supplement: Supplementary file 1 [file ijms-25-11847-s001.zip › SupplementaryTable_S8.docx]

**Table S8.** Study design diagram

| Study Component | Details |
| --- | --- |
| 1. Patient Selection Criteria | |
| Inclusion Criteria | Patients aged 65 and above  Undergoing cardiac surgery involving cardiopulmonary bypass (CPB) |
| Exclusion Criteria | Emergency intervention  Aortic surgery  Significant carotid artery stenosis  Parkinson's disease  Liver cirrhosis (Child-Pugh Class B or C)  Use of anticholinergic drugs, antidepressants, antiepileptic medications, or chemotherapy drugs |
| 2. Patient Recruitment | |
| Time Frame | June 2019 to January 2021 |
| Total Patients Enrolled | 39 |
| 3. Assessment of Postoperative Delirium (POD) | |
| Assessment Tool | Confusion Assessment Method for the Intensive Care Unit (CAM-ICU) |
| Initial Assessment | 6–8 hours post-surgery |
| Subsequent Assessments | Twice daily for 5 days |
| Patient Classification | POD Group: Patients with a positive CAM-ICU test at any time point (n = 12)  Non-POD Group: Patients without any positive CAM-ICU tests (n = 27) |
| 4. Blood Sample Collection and Preparation | |
| Timing | 24 hours after cardiac surgery |
| Procedure | Collection of venous blood samples using 9 ml BD Vacutainer® K2EDTA tubes |
| Plasma Separation | Centrifugation at 2000 g and 4°C for 15 minutes  Aliquoting and storage at -80°C |
| 5. Metabolomic Analysis | |
| Sample Preparation | Mixing 100 µl of plasma with 400 µl of cooled methanol/acetonitrile (1:1)  Vortexing and centrifugation at 16,000 rpm and +4°C for 15 minutes  Supernatant transfer to vial inserts for analysis |
| Quality Control | Preparation of two quality control samples by pooling plasma from POD and non-POD groups |
| Instrumentation | High-Performance Liquid Chromatography-Mass Spectrometry/Mass Spectrometry (HPLC-MS/MS)  Chromatograph: Shimadzu LC-20AD Prominence  Mass Spectrometer: 6500 QTRAP (AB SCIEX) |
| Data Acquisition | Detection in both positive and negative ion modes  Multiple Reaction Monitoring (MRM) mode  Collection and processing using Analyst 1.6.2 and MultiQuant 2.1 software  Peak area values obtained for statistical analysis |
| 6. Data Preprocessing | |
| Missing Value Imputation | If missing values ≤5% of total, replace with the median of observed values |
| Data Transformation | Log-transformation of metabolite concentration values |
| 7. Statistical Analysis | |
| Univariate Analysis | Mann-Whitney U test to compare metabolite levels between POD and non-POD groups |
| Multiple Testing Correction | Benjamini-Yekutieli procedure to control the False Discovery Rate (FDR) |
| 8. Machine Learning Approaches | |
| Genetic Algorithm (GA) | Implemented using the PyGAD Python module  Objective: Identify combinations of metabolites that best differentiate POD and non-POD groups |
| Fitness Function | Construct an XGBoost classification model using selected metabolites  Evaluate minimum accuracy across 5-fold cross-validation |
| Denoising Autoencoder (DAE) | Implemented using PyTorch  Architecture:  - Three fully connected layers (input, hidden, output) with ReLU activation  - Input/Output Layers: 210 neurons (number of metabolites)  - Hidden Layer: 50 to 149 neurons  Training:  - Optimizer: Adam (learning rate = 0.001)  - Epochs: 20; Batch Size: 8  - Loss Function: Mean Squared Error (MSE)  - Data Splitting: 80% training, 20% testing  Objective: Detect anomalies in metabolomic profiles to identify secondary metabolic markers |
| 9. Metabolic Pathway Enrichment Analysis | |
| Tool Used | MetaboAnalyst 5.0 |
| Methodology | Perform enrichment analysis to identify overrepresented KEGG metabolic pathways  Input lists of significant metabolites from statistical and machine learning analyses |
| 10. Gene Network Reconstruction | |
| Tool Used | ANDVisio (part of the ANDSystem) |
| Objective | Reconstruct molecular genetic pathways involving enzymes from identified metabolic pathways  Explore regulatory interactions between POD genetic markers and metabolic pathway enzymes |
| Approach | Use predefined templates to build regulatory pathways considering:  - Protein-protein interactions  - Protein function regulation  - Gene expression regulation  - Combined regulatory pathways |
